# A Predictive Model of Perceived Susceptibility during the Year before Coronary Artery Bypass Grafting

**Published:** 2018-01

**Authors:** Mozhgan Saeidi, Saeid Komasi

**Affiliations:** 1 *Cardiac Rehabilitation Center, Imam Ali Hospital, Kermanshah University of Medical Sciences, Kermanshah, Iran. *; 2 *Clinical Research Development Center, Imam Reza Hospital, Kermanshah University of Medical Sciences, Kermanshah, Iran.*

**Keywords:** *Coronary artery bypass*, *Perception*, *Risk factors*

## Abstract

**Background: **Based on the protective health model, one of the most important components of etiological factors leading to protective health behaviors is perceived risk or perceived susceptibility. Accordingly, the present study was conducted to assess the uncontrolled and controlled effects of some factors in predicting perceived susceptibility among coronary artery bypass graft (CABG) patients.

**Methods: **The data for the present cross-sectional study were gathered via assessment of 1052 CABG patients who referred to an outpatient cardiac rehabilitation clinic in a hospital in Iran between 2010 and 2014. The patients completed a checklist containing demographics, risk factors, and a single closed-ended question regarding perceived susceptibility at the beginning of their rehabilitation program. Binary logistic regression analysis was applied to identify the demographic and clinical correlations related to perceived susceptibility.

**Results: **Totally, 776 (73.8%) of the 1052 participants were male. The mean age of the patients was 58.0 ± 9.1 years. The results revealed that only 13.7% of the patients had perceived susceptibility; in addition, higher age (p value = 0.003) and family history of cardiac diseases (p value = 0.001) were able to significantly predict perceived susceptibility. When the demographic variables were controlled, once again age and family history of cardiac diseases were able to significantly increase perceived susceptibility by approximately 1.04 and 29.6 times, respectively.

**Conclusion: **Our results revealed that higher age and family history of cardiac diseases were able to significantly predict perceived susceptibility among our CABG patients.

## Introduction

Cardiovascular diseases (CVDs) as one of the chronic medical situations are known as the main cause of death in the United States of America and other industrial countries. They account for approximately one-third of the mortality rate among patients.^1^ In Iran, approximately 15 million persons suffer from CVDs and millions are at risk.^2^ CVDs have dangerous consequences, and a large number of Iranian cardiac patients die as a result of these diseases.^3^

Nowadays, progressively more people are at risk of CVDs and they are not well informed about it.^4^ These risk factors comprise biological, environmental, physiological, behavioral, and psychological components.^4^ Further, it seems that people’s knowledge on CVDs is poor, especially at-risk individuals, such that a significant number of cardiac patients are not well informed about the cause of their illness.^2^^, ^^4^ Moreover, there is no relationship between perceived risk factors by patients and actual risk factors in most cases.^5^ This issue indicates that most people do not have the correct perception about the nature of CVDs and their risk factors.^6^

Based on the protective health model, one of the most important components among etiological factors leading to protective health behaviors is perceived risk or perceived susceptibility.^7^ Perceived susceptibility, also called “perceived vulnerability”, refers to one’s perception of the risk or the chances of contracting a health disease or condition.^8^ Perceived susceptibility is one of many direct causes of behavior and a distal cause of behavior that operates through its influence on more proximal determinants.^9^ Risk perception, worry, severity, and control constitute the integral part of theories that explain health behavior such as the health belief model,^10^ common-sense model,^11^ and theory of planned behavior.^12^ Based on perceived susceptibility, Gerend et al.^7^ suggested that individuals exclusively attend to their actual risk factors when they know that they are at risk of disease and they have true knowledge about the risk factors. The role of risk perception in decision-making and health behaviors is approved through the results of a meta-analysis on tested evidence.^13^

Studies have been previously carried out on effective variables in risk and perceived susceptibility in chronic diseases and controversies. Based on the results, factors such as family medical history,^14^^, ^^15^ gender,^14^ age,^16^ and actual risk factors^17^^, ^^18^ are effective in perceived susceptibility. Additionally, previous research has indicated that there is no relationship between perceived risk and actual risk factors and demographic variables or that there is a very weak relationship between the mentioned factors.^19^^, ^^20^ Despite such controversies, there have been only a few studies on perceived susceptibility among cardiovascular patients carried out in Iran. Although marked developments have been made in diagnosis and treatment,^21^ it is clear that primary prevention has more importance in saving financial and human resources and that it needs self-care behaviors and healthy lifestyle, which are affected by individuals’ knowledge on susceptibility and risk perception. 

The importance of perceived susceptibility in primary prevention has led to the identification of its correlations among cardiovascular patients. There are 2 goals through the assessment of demographic and clinical variables such as the actual risk factors of cardiovascular patients: to examine the uncontrolled effect of each variable in predicting perceived susceptibility and to assess the controlled effect of each variable in predicting perceived susceptibility among coronary artery bypass graft (CABG) patients.

## Methods

In the present cross-sectional study, cardiovascular post-CABG patients who referred to the Center for Outpatient Cardiac Rehabilitation (CR) of Imam Ali Hospital, in the Iranian city of Kermanshah, between January 2010 and January 2014 were asked to participate in this research. Kermanshah City is the center of Kermanshah Province in the western part of Iran and it is about 326 miles from Tehran (Capital of Iran). Based on the 2011 census, the population of this city was estimated at 851 405 people.^22^ Imam Ali Hospital in Kermanshah is a governmental hospital with 214 beds. 

The inclusion criteria consisted of ability to establish and maintain appropriate interpersonal relationships, age between 30 and 80 years, absence of psychiatric medications, absence of a personal history of CVDs, and willingness for participation. The exclusion criterion was the presence of mismatch between the self-reported data and the medical record.

Patients referring to Imam Ali Hospital are from western Iranian provinces, including Kermanshah, Ilam, Kurdistan, Lorestan, and Hamadan, as well as the adjacent counties of these provinces. Fifty-six patients were excluded because they did not meet the inclusion criteria; the sample size, thus, comprised 1052 CABG patients who had registered in an outpatient CR program (approximately 40% of all CABG patients) from January 2010 to January 2014. 

The patients who gave their consent to participate in the study were referred to the research team by a supervising nurse and a clinical psychologist in the CR program. The research team informed the patients about the confidentiality of this research, and demographic characteristics such as risk factors and perceived susceptibility were gathered via appropriate instruments. First, a clinical psychologist asked and recorded the patients’ self-reported demographics and perceived susceptibility. Thereafter, a cardiologist, a nutritionist, a clinical psychologist, and a matron of the ward interviewed the patients separately and recorded their medical history and clinical conditions in a checklist containing demographics and risk factors. Finally, the demographic data and the medical risk factors were matched and verified through medical records audit.

Information regarding self-reported age, gender, marital status, educational level, and occupation was collected in the baseline survey. Family history of CVDs and risk factors including active smoking, passive smoking, substance addiction, previous history of hypertension, diabetes history, and hyperlipidemia history were classified based on the participants’ reports. Furthermore, medical risk factors such as hypertension, diabetes, and hyperlipidemia were matched and verified via medical records audit by the hospital research personnel. All the data were registered in the demographic and risk-factor checklist. The data were utilized to calculate actual cardiovascular risk (1 = presence of risk and 0 = absence of risk): high blood pressure, diabetes, high cholesterol, cigarette smoking, passive smoking, drug addiction, previous history of CVDs, and family history of CVDs.

Perceived susceptibility (perceived risk or perceived vulnerability) refers to one’s perception of the risk or the chances of contracting a health disease or condition such as CVDs.^8^ There is a wide range of opinions among individuals about personal susceptibility to a disease. The range of opinions includes total denial of the possibility of contracting a condition; admission to a possibility that the disease may occur, but not to them; and admission to a belief of actual danger.^23^ There are different instruments and methods for measuring perceived susceptibility and perceived risk.^24^^-^^26^ However, the factor in this research was a twofold criterion variable. The researchers applied a closed-answer single item “Were you expecting cardiac disease in the year before surgery?” The answers were registered as yes/no. Our goal was to find out whether the patients expected to have heart disease before surgery (i.e., when they were healthy). We were inspired by the studies of Andersen et al.^27^ and Wang et al.^14^ in the evaluation of severity, worry, and perceived control.

The patients’ characteristics with and without perceived susceptibility were compared via the χ^2 ^tests for the nominal and classified variables and the analysis of variance (ANOVA) for the continuous variables. A binary logistic regression analysis was applied to identify the demographic and clinical correlations related to perceived susceptibility. Perceived susceptibility (yes/no) was entered into the analysis as a criterion variable, and the demographic and clinical factors were the predictor variables. The predictor variables, comprised of gender, age, marital status, education level, and job, and risk factors, consisting of diabetes, hypertension, hyperlipidemia, family history, cigarette smoking, passive smoking, and substance abuse, were entered in the analysis. Before the analysis, the necessary statistical presumptions for the binary logistic regression analysis were assessed. The analyses were carried out using SPSS, version 20, and p value less than 0.05 was considered statistically significant.

## Results

Among the 1052 (73.8% male) patients who participated in the CR program, 144 (13.7%) patients had perceived susceptibility. The clinical and demographic characteristics of the samples are presented generally and separately in Table 1. As can be seen in the table, there was a difference between the 2 groups in terms of family history of CVDs and diabetes, indicating that those with a family history of the disease had significantly more perceived susceptibility in the year before surgery. Moreover, those with diabetes history had significantly less perceived susceptibility than those without it.

The Hosmer–Lemeshow test indicated that the fit of the model was appropriate and our model correctly predicted 90.1% membership of the group. Overall, the model was statistically significant. The indicators of the effect size demonstrated a suitable explanatory power with respect to perceived susceptibility, suggesting that our model was able to explain 23.4% to 42.6% variance of perceived susceptibility.

The adjusted odds ratios, 95% confidence intervals, and p values for each covariate are presented in Table 2. In relation to the regression model, the 2 variables were found to be independently and significantly associated with perceived susceptibility. According to the results, older people and people with a family history of cardiac diseases had more possibility of predicting a cardiac event in the year before surgery than the others. Specifically, older age and family history were able to increase perceived susceptibility by about 1.04 and 29.6 times respectively.

In Table 3, adjustment for the demographic variables was conducted and the significance of each variable was assessed in 4 models. In Model 1, after adjustment for age and gender, it was indicated that age and family history were able to predict perceived susceptibility. In Model 2, after adjustment for age, gender, and educational level, once again it was indicated that age and family history were able to predict perceived susceptibility. In Model 3, after adjustment for age, gender, educational level, and marital status, age and family history were significant. Finally, in Model 4, after adjustment for age and gender, educational level, marital status, and job, once again it was indicated that age and family history were able to predict perceived susceptibility.

**Table 1:** Baseline demographic and clinical characteristics in the overall population and in those with and without perceived susceptibility*

Characteristic			Overall Population (n=1052)		Without Perceived Susceptibility (n=908)		With Perceived Susceptibility (n=144)		P value
Sex									0.511
Male Female	776 (73.8) 276 (26.2)			780 (74.1) 272 (25.9)		752 (71.5) 300 (28.5)			
Age (y)			58.0±9.1		57.9±9.1		58.6±8.9		0.410
Education attainment									0.667
Illiterate < 9 years < 12 years 12 years 14 years 16 years > 16 years	383 (36.3) 331 (31.5) 148 (14.1) 150 (14.3) 19 (1.8) 18 (1.7) 3 (0.3)			389 (37.0) 335 (31.8) 143 (13.6) 147 (14.0) 17 (1.6) 16 (1.5) 5 (0.5)		336 (31.9) 306 (29.1) 176 (16.7) 176 (16.7) 29 (2.8) 29 (2.8) 0			
Marital status									0.704
Married Single, Divorced, or Separated		963 (91.5) 89 (8.5)		962 (91.4) 90 (8.6)		972 (92.4) 80 (7.6)			
Occupation									0.871
Marketing Housewife Retired Employee	400 (38.0) 250 (23.8) 286 (27.2) 116 (11.0)			404 (38.4) 248 (23.6) 283 (26.9) 117 (11.1)		373 (35.4) 263 (25.0) 307 (29.2) 109 (10.4)			
Risk factors history									
Diabetes Hypertension Hyperlipidemia Family history Smoking Passive smoking Drug addiction	389 (37.0) 350 (33.3) 305 (29.0) 201 (19.1) 428 (40.7) 89 (8.5) 195 (18.5)			406 (38.6) 345 (32.8) 309 (29.4) 108 (10.3) 423 (40.2) 85 (8.1) 194 (18.4)		278 (26.4) 380 (36.1) 278 (26.4) 782 (74.3) 460 (43.7) 109 (10.4) 204 (19.4)		0.005 0.445 0.465 0.001 0.425 0.362 0.769	

* Data are presented as mean±SD or n (%)

**Table 2:** Predictors of perceived susceptibility in the study population

Characteristic				Perceived Susceptibility (%)				OR (95% CI)				P value
Sex												0.755
Male Female		13.3 14.8			Reference 1.25 (0.31-5.00)							
Age(y)				-				1.04 (1.01-1.08)				0.003
Education attainment												
Illiterate < 9 years < 12 years 12 years 14 years 16 years > 16 years		12.0 12.7 16.2 15.9 21.0 22.2 0			Reference 1.44 (0.77-2.67) 1.82 (0.78-4.23) 1.72 (0.73-4.08) 1.23 (0.28-5.47) 1.49 (0.30-7.26) 5.43 (0.07-70.91)				0.252 0.170 0.226 0.788 0.626 0.999			
Marital status												0.431
Married Single, Divorced, or Separated			13.8 12.3			Reference 0.70 (0.29-1.70)						
Occupation												
Marketing Housewife Retired Employee	12.7 14.4 14.7 12.9						Reference 1.21 (0.28-5.17) 0.75 (0.39-1.45) 0.55 (0.23-1.30)				0.799 0.395 0.170	
Cardiac risk factors												
Diabetes Hypertension Hyperlipidemia Family history Smoking history Passive smoke history Drug addiction history			9.8 14.8 12.4 53.2 14.7 16.8 14.3			0.63 (0.38-1.04) 1.08 (0.67-1.74) 0.99 (0.60-1.64) 29.62 (18.63-49.00) 1.55 (0.93-2.58) 1.31 (0.56-3.08) 1.21 (0.66-2.24)				0.071 0.744 0.976 0.001 0.091 0.533 0.545		

**Table 3:** Predictors of perceived susceptibility after adjustment for the demographic characteristics

	Model 1		Model 2		Model 3		Model 4	
	AOR	95% CI	AOR	95% CI	AOR	95% CI	AOR	95% CI
Age	1.04	1.01-1.08	1.04	1.01-1.07	1.04	1.01-1.07	1.04	1.01-1.07
Family history	29.63	18.62-47.12	29.90	18.72-47.71	29.50	18.52-47.01	29.54	18.50-47.00

Model 1, Adjusted for age and sex; Model 2, Adjusted for age, sex, and education; Model 3, Adjusted for age, sex, education, and marital status; Model 4, Adjusted for age, sex, education, marital status, and occupation

## Discussion

The aim of the present study was to assess the predictors of perceived susceptibility toward CVDs in CABG patients. The results showed that only 13.7% of the patients had expected cardiac diseases in the year before surgery. This finding is consistent with past studies that suggest there are incorrect beliefs and poor perceived risk in general and clinical populations.^19^^, ^^20^^, ^^28^ It seems that patients and at-risk people do not have appropriate estimations about their conditions and they have a type of incorrect optimism such that approximately 40% of the general population underestimate the possibility of their CVDs.^29^

According to some studies,^16^^, ^^28^^, ^^30^ older people predict cardiac events in the year before surgery more than other patients. Age as a biological marker has more effect on the disease process among the nonspecific parameters of cardiac diseases such that the risk of the presentation and confirmation of CVDs increases with age.^31^ In addition, physiological risk factors such as hypertension and diabetes increase with age and these risk factors increase worry and risk perception. 

Furthermore, after controlling for all the demographic variables, we found that the patients with a family history of cardiac diseases had higher perceived risk than the others and that they predicted this event with higher possibility. In fact, this factor was able to increase perceived risk to about 29.6-fold, which is in accordance with past studies.^14^^, ^^15^^, ^^30^ The results of qualitative studies indicate that most cardiovascular patients know that family history is one of the major risk factors for their disease.^32^^, ^^33^ The uncontrollable nature of biological and hereditary disease, based on these patients’ opinions, makes them aware that they are at greater risk of future cardiac events^34^ and they have higher perceived risk. According to a report by Saeidi et al.,^2^ the main perceived risk factor for CVDs, especially in younger patients, is a positive family history. Also in our study, most of the patients were younger than 55 years; consequently, the relationship between perceived susceptibility and family history can be explained. 

Finally, our results showed that none of the actual risk factors such as histories of hypertension, diabetes, hyperlipidemia, smoking, substance abuse, and passive smoking could predict perceived risk. This may be a consequence of poor health literacy present in the general population and at-risk individuals. About 36% of our patients were illiterate and 82% of them had an educational level less than a high school diploma. Poor health literacy is related to poor health self-reports, inadequate health knowledge, poor self-management skills, and inappropriate health choices.^29^ In this regard, the results of a study indicated that only 35% of diabetic patients know that they are at risk of CVDs.^32^ In addition, although smokers know that they are at more risk than nonsmokers, they have more optimistic bias than nonsmokers.^35^

The current study has several strengths and limitations. One of these strengths is the selection of variables which can explain 23.4% to 42.6% variance of perceived susceptibility at the same time. The appropriate sample size according to patients’ information with medical records can be considered another strong point of the study. We studied only CABG patients in a hospital in the western part of Iran. In addition, we only assessed the patients who were referred to outpatient CR and the majority of the patients (approximately 60% of all the population) were not assessed because they did not refer to this center. We recommend that future studies be carried out with a randomized selection of hospitals from different parts of Iran for proper generalization of data and that all CABG patients be enrolled in these studies before they are released. Due to the unavailability of these patients’ medical histories and psychological conditions before CABG, these variables were excluded in our analysis. Another important limitation of our study is the lack of access to the histories of other symptomatic disorders like osteoarthritis, respiratory problems, and psychiatric disorders. Consideration of patients’ medical histories may increase the accuracy of future studies. In addition, given that stress is associated with many life events such as job status and comorbidities like diabetes and hypertension, we recommend that these be assessed in future studies. The risk of recall bias is another limitation because of the wide range of the time allocated for the inclusion of patients (2010–1014). Finally, although only a closed-answer single item was applied in some studies for the evaluation of perceived susceptibility,^13^^, ^^26^ using standard instruments can reduce bias in results.

## Conclusion

The results of the current study demonstrated that 86.3% of the study population with established heart diseases, even during the year before CABG, had significantly underestimated the risk of deadly diseases. The results revealed that only 13.7% of the patients had perceived susceptibility and higher age and family history of cardiac diseases were able to significantly predict perceived susceptibility. Although older people and patients with a family history of cardiac diseases have a suitable understanding of the risk, other traditional risk factors do not play a role in alerting the population susceptible to the disease. It seems necessary that appropriate training about the traditional risk factors of CVDs be provided to the at-risk population in the western part of Iran. 

## Notes:

***This paper should be cited as:*** Saeidi M, Komasi S. A Predictive Model of Perceived Susceptibility during the Year before Coronary Artery Bypass Grafting. J Teh Univ Heart Ctr 2018;13(1):6-12.
